# Informing Patients with Acute Stroke About their Risk of Dementia: A Survey of UK Healthcare Professionals

**DOI:** 10.1016/j.jstrokecerebrovasdis.2021.106279

**Published:** 2022-03

**Authors:** Emily L. Ball, Gillian E. Mead, Eugene Y.H. Tang, Dorota Religa, Terence J. Quinn, Susan D. Shenkin

**Affiliations:** a Centre for Clinical Brain Sciences, University of Edinburgh, Scotland, UK; b Geriatric Medicine, Usher Institute, University of Edinburgh, Scotland, UK; c Population Health Sciences Institute, Newcastle University, England; d Division of Clinical Geriatrics, Karolinska Institutet, Stockholm, Sweden; e Institute of Cardiovascular and Medical Sciences, University of Glasgow, Scotland, UK

**Keywords:** (between 4 and 8), Stroke, Dementia, Cognitive decline, Post-stroke dementia, Post-stroke cognitive impairment, Prognostic factors

## Abstract

**Objectives:**

Cognitive problems following stroke are of key concern to stroke survivors. Discussing risk of dementia at the time of stroke could have implications for follow-up care. However, informing someone who has just had a stroke about risk of dementia could cause distress. This survey explored healthcare professionals’ views on discussing risk of post-stroke dementia at the time of stroke.

**Materials and methods:**

This online survey was aimed at all UK healthcare professionals who care for patients with stroke. The survey was distributed via the mailing lists of seven professional stroke-related organisations and Twitter. Descriptive statistics were used to summarise findings.

**Results:**

Sixty healthcare professionals completed the survey. Healthcare professionals were aware of the main risk factors associated with post-stroke dementia (e.g. previous stroke, age). Most respondents (N=34/60, 57%) thought that patients with acute stroke would benefit from knowing if they are at high risk of dementia, and 75% (N=45/60) agreed that carers would benefit. Despite this, the majority of healthcare professionals (N=47/53, 89%) who cared for patients with acute stroke in the past year said they rarely/never discussed dementia with their patients. Most respondents (N=46/60, 77%) thought risk of dementia should be discussed 1-6 months post-stroke.

**Conclusion:**

Although healthcare professionals felt it would be helpful to discuss risk of post-stroke dementia, in practice, most said that they rarely or never discussed this with their patients. Stroke survivors could benefit from a healthcare system that offers appropriate follow-up care and support to patients at high risk of dementia.

## Highlights

• Healthcare professionals (HCPs) are generally aware of risk factors for post-stroke dementia.
• HCPs do not routinely discuss dementia risk with patients at time of stroke.
• HCPs said carers were more likely than patients to ask about risk of dementia.
• HCPs think it could be helpful to discuss risk of dementia at the time of stroke.
• HCPs think that 1-6 months after stroke was the best time to discuss risk of dementia.

## Introduction

People who have a stroke are at an increased risk of developing dementia.^1^^,^^2^ According to the 2021 James Lind Alliance–Stroke Association Priority Setting Partnership, cognitive issues after stroke are a key concern of people who have a stroke.^3^ Identifying which patients with acute stroke are at risk of dementia could help patients and their carers to plan for the future. However, informing a patient who has just had a stroke about their risk of dementia may cause anxiety.

### Risk factors for post-stroke dementia

Post-stroke dementia involves a diagnosis of any type of dementia following a stroke.^4^ Key risk factors associated with the development of post-stroke dementia include: recurrent stroke, low education, older age, diabetes mellitus, atrial fibrillation, and neuroimaging features suggesting pre-existing neurodegeneration including atrophy and white matter lesions.^2^^,^^5^^,^^6^ Modifying the risk factors for dementia, including preventing recurrent strokes, could reduce the risk of developing post-stroke dementia.^1^ Medical history and computed tomography (CT) brain images are routinely collected at the time of stroke. Healthcare professionals may be able to use this information to identify which patients with acute stroke are at risk of developing dementia and provide appropriate follow-up, and potentially implement strategies to reduce the risk of dementia.

### Predicting post-stroke dementia

Might a tool for prediction of post-stroke dementia be useful in clinical practice? There are currently no robust prognostic tools that accurately predict which stroke patients are at risk of developing post-stroke dementia.^7^ A qualitative study used semi-structured interviews to assess the acceptability to patients, carers, and clinicians of using dementia risk prediction tools as part of post-stroke follow-up care.^8^ This study interviewed 15 stroke patients and their carers (at six and twelve months post-stroke) and 17 clinicians, in the North East of England. Although some stroke survivors thought that knowing their risk of dementia may allow them to prepare for a dementia diagnosis, they were concerned about the anxiety this may cause. Clinicians also raised concerns about whether a risk assessment tool for post-stroke dementia would make a difference to the standard care received by stroke patients.

### Care-pathway after stroke

National Institute for Health and Care Excellence guidelines recommend follow-up appointments at six months, twelve months, and then annually after stroke.^9^ However, according to the Sentinel Stroke National Audit Programme, less than half of people who had a stroke in 2019 to 2020 received a follow-up appointment at six months in England, Wales, and Northern Ireland.^10^ All four counties within the UK are striving to better support patients after stroke, but implementation of current guidelines is still far from universal.^11^, ^12^, ^13^, ^14^

People who have had a stroke have reported challenges with accessing support.^15^ A survey of 1421 stroke survivors in England reported that 45% of people who had a stroke felt abandoned when they left hospital, and that access to post-acute care varied across the country.^16^ Tang *et al's* qualitative study found that healthcare professionals placed more focus on post-stroke physical recovery, had challenges with starting a discussion about memory issues, and reported that there was an unclear service pathway for patients presenting with cognitive issues following a stroke.^17^ Patients and caregivers also reported that memory problems after a stroke negatively affected their daily life and emotions.^18^ If risk of future cognitive problems are not identified during the acute hospital stay they may not come to medical attention until there is a crisis.

### Receiving a diagnosis of dementia

Coping with symptoms of dementia can be challenging. On average, the length of time for a person seeking help for a memory problem is 2.5 years from first recognising symptoms.^19^ Older people and stroke survivors have reported that they are fearful of receiving a dementia diagnosis.^15^^,^^20^ Undergoing cognitive assessments and receiving a diagnosis of dementia can also be stressful. A qualitative study conducted in Sweden interviewed 18 patients who underwent cognitive assessments following referral from their primary care provider (not in the context of stroke). Patients reported that they did not understand the process of a cognitive assessment or the possible outcomes, they had concerns about living with a neurocognitive disorder, and reported feelings of shock and violation following the assessment.^21^

### Rationale

Previous research has focused on predicting and preventing dementia following a stroke, yet no studies have explored whether it would be acceptable to discuss risk of dementia at the time of stroke.^1^^,^^7^^,^^22^ Discussing risk of dementia at the time of stroke, while the patient is receiving medical help, may be an ideal time to educate patients about the symptoms of dementia, inform patients about how to modify lifestyle risk factors for dementia, and highlight the support that is available. However, informing people who have just had a stroke that they also have an increased risk of developing dementia, a condition that is not currently curable, may cause emotional distress and potentially, interfere with optimal cognitive and physical recovery.

### Aims

This UK-wide quantitative survey aimed to explore healthcare professionals’ views on discussing risk of post-stroke dementia at the time of stroke. This survey aimed to explore UK healthcare professionals’ views on: (1) identifying patients with acute stroke who are at risk of dementia, (2) informing patients with acute stroke and their caregivers about risk of dementia, and (3) the use of a tool for predicting post-stroke dementia in clinical practice.

## Materials and methods

### Study design

This cross-sectional survey is reported according to the Checklist for Reporting Of Survey Studies (CROSS) guidelines.^23^

### Sample characteristics

This online survey was aimed at all UK healthcare professionals who care for patients with stroke, including physicians, nurses, and allied health professionals.

### Survey structure

A copy of the survey is provided in Appendix 1. As we wanted to seek the views of all UK healthcare professionals who care for patients with stroke, even if they do not currently care for patients with acute stroke, we included self-report questions and scenario-based questions and asked them to imagine they are caring for a patient with an acute stroke.

The survey included questions relating to respondent characteristics (country of work, profession, length of time working with patients with stroke, healthcare setting) and addressed seven key areas (Table 1).

**Table 1:** Key areas addressed in questionnaire.

1. Do healthcare professionals think patients with acute stroke, and their carers, are likely to benefit from knowing the risk of post-stroke dementia?
2. Are patients with acute stroke routinely informed about their risk of post-stroke dementia?
3. Does the severity of stroke influence which patients with acute stroke are informed about risk of post-stroke dementia?
4. Are healthcare professionals aware of the risk factors associated with post-stroke dementia?
5. Who should inform patients with stroke about their risk of post-stroke dementia?
6. When should patients with stroke be informed about their risk of post-stroke dementia?
7. At what level of risk would healthcare professionals discuss risk of post-stroke dementia with a patient with acute stroke and at what level of risk would healthcare professionals inform the patient's GP about risk of post-stroke dementia?

### Survey responses

Responses were anonymous. Participants had to provide a response to each question. The survey used a combination of multiple-choice questions, Likert-type scales (frequency, likelihood, level of agreement), and rating scales. Respondents were able to provide free text responses to expand on their answers. We also embedded internal checks within our survey. When respondents were asked to identify risk factors for post-stroke dementia, they were provided with a list of known risk factors, which also included irrelevant risk factors (e.g. asthma, cancer, tumour).

### Piloting

The survey was piloted on five healthcare professionals (occupational therapist, physiotherapist, clinical psychologist, speech and language therapist, geriatrician) who provided feedback on the clarity of the questions, and the layout of the survey. Revisions were made prior to distributing the survey.

### Survey administration

The survey was created and distributed using Jisc Online Surveys.^24^ Seven professional stroke-related organisations agreed to distribute the survey (Contact Help and Advice Information Network (CHAIN) Stroke Specialist Interest Group, Organisation for Psychological Research Into Stroke, British Association of Stroke Physicians, British Geriatrics Society – Dementia and Related Disorders Specialist Interest Group, National Stroke Nursing Forum, Scottish Stroke Nurse Forum, and Royal College of Speech and Language Therapists – Clinical Excellence Networks focussed in stroke and brain injury). The British Association of Stroke Physicians sent a reminder to their mailing list two months after the survey was initially distributed. Between November 2020 and January 2021, the organisations either emailed their members with the invitation email and link to the survey, or included information about the survey in their newsletter. Due to a low responses rate (N=52), we decided, post-hoc, to distribute the survey via Twitter in March 2021, and the survey remained open for six weeks (N=8).

### Definitions

We defined the following terms in the survey: (1) stroke: any type of stroke (not including transient ischemic attack). (2) Acute stroke period: within two weeks of a stroke. (3) Post-stroke dementia: persistent, worsening cognitive problems that interfere with daily activities, this can be any type of dementia diagnosed after a stroke. (4) A risk prediction tool for post-stroke dementia: a tool that can be used to calculate the probability or risk of a person with acute stroke developing dementia. We also used the phrase ‘continuous decline in cognition’ to understand whether healthcare professionals discussed the symptoms of dementia without using the diagnostic term ‘dementia’.

### Ethical considerations

Ethical approval was obtained from the College of Medicine and Veterinary Medicine Ethics Committee, University of Edinburgh (20-EMREC-004 SA01, approved 25 September 2020). Sponsorship was gained from the University of Edinburgh Academic and Clinical Central Office for Research and Development (ACCORD).

### Data analysis

Data were analysed using Jisc Online Surveys and figures were created using R 4.1.0.^24^^,^^25^ We used descriptive statistics (percentages, rounded to whole numbers) to summarise these data.

We categorised professions into three categories (physicians, nurses, allied health professionals). We performed a sensitivity analysis to assess whether profession of the respondent influenced who they thought should inform patients about their risk of dementia. Respondents were asked where they usually work with stroke patients and could select from a list of options (Stroke unit, Acute assessment ward, Emergency Department, GP Practice), or provide a free text response.

### Free text responses

We used an inductive coding approach to code free text responses.^26^ When all responses had been collected, ELB (non-clinician researcher) read the free text responses in detail and identified five subcategories (Table 2), a clinician confirmed the coding (SDS). We assigned relevant quotes from each free text response into one or more of these subcategories and narratively summarised the key themes. Quotes from the free text are provided in Appendix 9-13.

**Table 2:** Free text responses were categorised into the following subcategories

• Focus of acute stroke discussions • Factors that would influence whether to discuss dementia at the time of stroke • Patient/carer related factors • Post-stroke care-pathway • Risk prediction tool

## Results

### Participants

Sixty healthcare professionals completed the survey (Table 3), including: physicians (N=31/60, 52%), allied health professionals (N=20/60, 33%), and nurses (N=9/60, 15%).

**Table 3:** Respondent characteristics.

	N (%)
**Country**	
Scotland	14 (23)
England	38 (63)
Northern Ireland	6 (10)
Wales	2 (3)
**Profession**	
Physician	31 (52)
Allied health professional	20 (33)
Nurse	9 (15)
**Length of time caring for patients with stroke**	
Less than 1 year	2 (3)
1-5 years	17 (28)
6-10 years	10 (17)
11-15 years	13 (22)
16-20 years	11 (18)
21+ years	7 (12)
**Healthcare setting (select all options that apply)**^a^	
Stroke unit	34 (57)
Acute assessment ward	17 (28)
Emergency Department	8 (13)
GP Practice	3 (5)
Other^b^	21 (35)

a Total percentage does not equal zero because respondents could select more than one option

b Other responses included: Community, Outpatients, Rehabilitation Unit, Older Adults Ward, Early Supported Discharge Service

### (1) Do healthcare professionals think patients with acute stroke, and their carers, are likely to benefit from knowing the risk of post-stroke dementia?

*Question: Imagine that you are able to identify which patients you think are at high risk of developing dementia within the next year. Please respond to the following statements regarding patients with acute stroke (Response options: Strongly agree, Agree, Neither agree nor disagree, Disagree, Strongly disagree):*

(1) *I think the patient would benefit from knowing they are at high risk of post-stroke dementia*
(2) *I think the patient's family/carers would benefit from knowing the patient is at high risk of post-stroke dementia*

Healthcare professionals thought that both patients and relatives were likely to benefit from knowing they are at high risk of post-stroke dementia, with 57% (N=34/60) agreeing that patients would benefit, and 75% (N=45/60) agreeing that relatives would benefit (Appendix 2).

### (2) Are patients with acute stroke routinely informed about their risk of post-stroke dementia?

*Please complete the following statement (select all options that apply). I discuss the risk of post-stroke dementia:*

(a) *With all patients with acute stroke*
(b) *With patients with acute stroke that I think have a high risk of developing dementia*
(c) *If the patient with acute stroke or their carer asks about post-stroke dementia*
(d) *I have never discussed post-stroke dementia with a patient with acute stroke*
(e) *Other (free text response)*

Thirty-seven percent (N=22/60) of healthcare professionals have never discussed post-stroke dementia with a patient with acute stroke (Appendix 3). Half of the respondents (N=30/60) said they would discuss risk of post-stroke dementia if the patient/carer asks, and only 12% (N=7/60) of healthcare professionals said they would discuss dementia if they thought the patient had a high risk of developing dementia. Only one healthcare professional said they discuss risk of post-stroke dementia with all patients with acute stroke.

*Question: Think about the patients with acute stroke that you have seen in the past year*

*(Response options: All of the time, Most of the time, Sometimes, Rarely, Never)*

(1) *How often did you discuss the risk of developing DEMENTIA with patients with acute stroke?*
(2) *How often did you discuss the risk of a CONTINUOUS DECLINE IN COGNITION with patients with acute stroke?*
(3) *How often did patients with acute stroke ask about the risk of developing DEMENTIA?*
(4) *How often did patients with acute stroke ask about the risk of a CONTINUOUS DECLINE IN COGNITION?*
(5) *How often did relatives/carers ask about the risk of the patient developing DEMENTIA?*
(6) *How often did relatives/carers ask about the risk of the patient having a CONTINUOUS DECLINE IN COGNITION?*

Of the healthcare professionals who cared for patients with acute stroke in the past year, 89% (N=47/53) said they rarely or never discussed dementia with their patients (Appendix 4).

Healthcare professionals reported they were more likely to discuss ‘continuous decline in cognition’ (sometimes = 26%, N=14/53), compared to ‘dementia’ (sometimes = 8%, N=4/53). Healthcare professionals reported that relatives/carers were more likely to ask about dementia or cognitive decline than patients were.

### (3) Does the severity of stroke influence which patients with acute stroke are informed about risk of post-stroke dementia?

*Question: How likely are you to discuss the risk of dementia with the following patients who had their stroke/TIA 5 days ago? (Response options: Extremely likely, Likely, Neutral, Unlikely, Extremely unlikely)*

(1) *A patient who had a TIA*
(2) *A patient who had a mild stroke (NIHSS 1-4)*
(3) *A patient who had a moderate stroke (NIHSS score 5-15)*
(4) *A patient who had a moderate to severe stroke (NIHSS 16-20)*
(5) *A patient who had a severe stroke (NIHSS 21-42)*

Healthcare professionals were more likely to discuss the risk of post-stroke dementia with patients with more severe presentations (Appendix 5). A majority of healthcare professionals (N=33/60, 55%) reported they were likely or extremely likely to discuss risk of dementia with patients who have had a moderate to severe stroke (NIHSS 16-20), or severe stroke (NIHSS 21-42), compared to only 22% (N=13/60) with patients who had a TIA, mild stroke or moderate stroke (NIHSS 1-15).

### (4) Are healthcare professionals aware of the risk factors associated with post-stroke dementia?

**Fig. 1:**
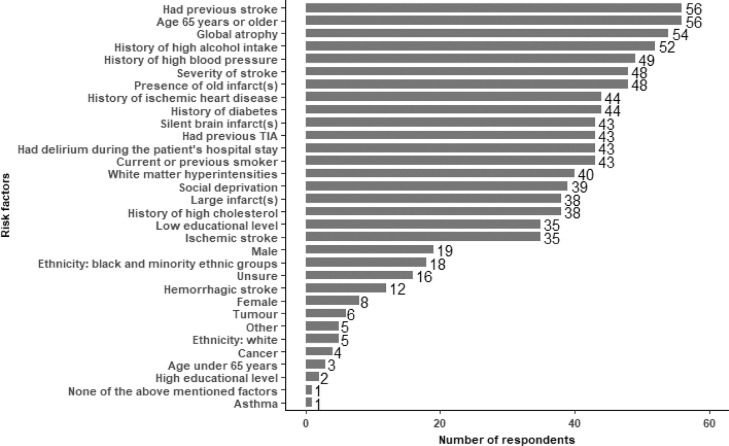
Question: Imagine that you see a patient who had a stroke 5 days ago. You are considering whether this patient is at risk of developing dementia within the next year. Which of these factors would make you concerned that this person has a high future risk of dementia? (Select all options that apply)

Healthcare professionals were aware of the main risk factors associated with post-stroke dementia (previous stroke, age, global atrophy) and correctly noted that asthma, cancer, tumour were not risk factors. Less than 10% (N≤6/60) of respondents selected these irrelevant risk factors (Fig. 1). There was uncertainty around stroke type and years of education of the patient. Respondents added other potential risk factors including: pre-stroke cognitive problems, cognitive problems at time of stroke, delirium during hospital stay, small vessel disease reported on radiology report, frailty, history of mental illness, vitamin B12 deficiency, and MTHFR polymorphism.

### (5) When should patients with stroke be informed about their risk of post-stroke dementia?

*Question: Imagine that you are able to identify which patients YOU think are at high risk of developing dementia within the next year. When would you choose to inform the stroke patient about their risk of post-stroke dementia?*

(a) *Within 2 weeks of their stroke*
(b) *1 month after their stroke*
(c) *3 months after their stroke*
(d) *6 months after their stroke*
(e) *I would not inform the patient*
(f) *Other (free text response)*

Most healthcare professionals felt that the most appropriate time to inform patients of their risk of dementia is between one to six months post-stroke (N=46/60, 77%) (Appendix 6). Very few respondents (N =3/60, 5%) would inform within two weeks and 8% (N=5/60) would not inform a patient with stroke.

### (6) Who should inform patients with acute stroke about their risk of post-stroke dementia?

*Question: Think about your answer to the previous question. At this point in time, who do you think should inform the patient about their risk of post-stroke dementia?*

(a) *Stroke physician*
(b) *General Practitioner*
(c) *Stroke specialist nurse*
(d) *Clinical psychologist or clinical neuropsychologist*
(e) *Occupational therapist*
(f) *Physiotherapist*
(g) *Speech and language therapist*
(h) *No one should inform the patient*
(i) *Other (free text response)*

When asked who should inform stroke patients about their risk of dementia, 53% (N=32/60) of healthcare professionals thought a stroke physician should inform the patient about risk of dementia. Only 12% (N=7/60) thought the patient's general practitioner (GP) should inform the patient (Appendix 7). Doctors were most likely to say that doctors (stroke physician or GP) should inform the patient (65%, N=20/31) but nurses and allied health professionals also felt that doctors were best placed to do this. Only 11% (N=1/9) of nurses thought a stroke specialist nurse should inform the patient, and 10% (N=2/20) of allied health professionals thought an allied health professionals should inform the patient.

*Question: Imagine that you are able to identify which patients you think are at high risk of developing dementia within the next year. Please respond to the following statements regarding patients with acute stroke. (Response options: Strongly agree, Agree, Neither agree nor disagree, Disagree, Strongly disagree)*

(1) *I would discuss the risk of dementia with the patient with acute stroke*
(2) *I would make the patient's GP aware that the patient is at high risk of post-stroke dementia*
(3) *I would feel confident discussing the risk of post-stroke dementia with the patient*

The majority of healthcare professionals would inform a patient's GP of the risk of post-stroke dementia (N=40/60, 87%) rather than discuss it with the patients themselves (N=29/60, 48%), with 45% (N=27/60) feeling confident to have this discussion (Appendix 8).

### (7) At what level of risk would healthcare professionals discuss risk of post-stroke dementia with a patient with acute stroke and at what level of risk would healthcare professionals inform the patient's GP about risk of post-stroke dementia?

*Question: Imagine that you see a patient who has had a stroke 5 days ago. You input the patient's information into a highly accurate risk prediction tool and calculate their risk of developing post-stroke dementia within the next year.*

(1) *At which level of risk would you discuss post-stroke dementia with the patient?*
(2) *At which level of risk would you make the patient's GP aware of the risk of post-stroke dementia?*

*(Response options: 0-20% risk of post-stroke dementia, 21-40% risk of post-stroke dementia, 41-60% risk of post-stroke dementia, 61-80% risk of post-stroke dementia, 81-100% risk of post-stroke dementia)*

**Fig. 2:**
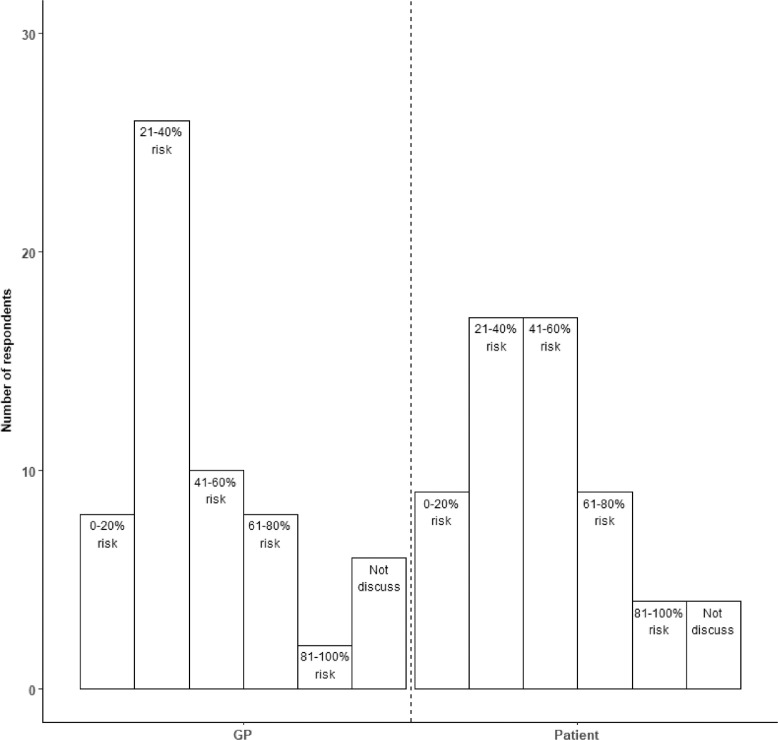
At which level of risk would you discuss post-stroke dementia with patients/their GP?

Although there was considerable variability in participant responses, the most frequent level of risk where respondents would discuss post-stroke dementia with the patient were 21-40% (N=17/60, 28%) and 41-60% (N=17/60, 28%) (Fig. 2). A majority of respondents would discuss risk with the patient's GP if the patient had a 21-40% (N=26/60, 43%) risk of developing post-stroke dementia within the next year.

### Free text responses

A narrative summary of responses are provided below. Quotes from respondents are provided in Appendix 9-13.

### Focus of acute stroke discussions in current clinical practice

Lots of information is given to patients and their carers following a stroke and healthcare professionals indicated that focus is often placed on survival, secondary prevention and rehabilitation. Healthcare professionals reported that patients and carers assume that the person will get better after a stroke, rather than worse. If cognition is mentioned, patients and carers tend to focus on acute stroke cognitive problems, which may improve. Several healthcare professionals thought it was too soon to discuss dementia at the time of the stroke.

### Factors that would influence whether healthcare professionals discuss dementia, at the time of stroke

Healthcare professionals said they would discuss risk of dementia, at the time of stroke, if the patient/caregiver initiated the conversation. They would also be more likely to discuss risk of dementia if the patient had delirium or cognitive problems at the time of stroke. Some healthcare professionals questioned whether it is beneficial to inform stroke patients about their risk of dementia if treatments are not available to prevent or delay the onset of dementia but highlighted that it may be helpful to educate patients about dementia and modifiable risk factors, providing this information be communicated in a positive way.

### Patient/carer related factors

Healthcare professionals highlighted that it is dependent on the individual patient, their ability to understand cognitive problems, and their readiness to know the information. Patients are given a lot of information and discussing risk of dementia soon after a stroke may cause distress and anxiety, therefore, it may be more appropriate to discuss at a follow-up appointment.

### Post-stroke care-pathway

Responses were varied regarding who should inform a patient with stroke about their risk of dementia: the stroke team, healthcare professionals already involved in the patient's care, healthcare professionals who have the knowledge to discuss risk of dementia, or a multidisciplinary team. Respondents highlighted that having a discussion about post-stroke cognitive problems involves complex conversations which may be best after discharge or during rehab, in a follow-up clinic, or during follow up year once they are home and starting to rebuild lives. Some respondents suggested that cognition should be assessed during follow-up to measure whether there is a deterioration. One respondent suggested that ‘we’ should screen for dementia in stroke.

### Using a risk prediction tool for post-stroke dementia

Some healthcare professionals thought that a prediction tool for post-stroke dementia would be helpful for discharge plans and planning follow-up care, providing that appropriate support mechanisms are available. However, they thought that it is important that using a prediction tool at the point of stroke should not deny access to treatments for those patients who have an increased risk of developing post-stroke dementia.

## Discussion

### Summary of findings

This survey of a multidisciplinary range of healthcare professionals found that although healthcare professionals felt it may be helpful to discuss the risk of post-stroke dementia at the time of stroke, in practice, most said that they rarely or never discussed this with their patients. The majority of healthcare professionals thought it would be better to discuss risk of dementia several months after stroke.

Our survey focused on seven key areas. We have summarised our findings to these questions here: (1) Healthcare professionals felt that it would be beneficial to discuss the risk of post-stroke dementia with patients and their carers at the time of stroke. (2) In practice, most healthcare professionals who care for patients with acute stroke said that they rarely or never discussed risk of dementia with their patients. (3) Healthcare professionals say they would be more likely to discuss risk of post-stroke dementia at the point of stroke if the patient had a more severe stroke (NIHSS 16-42). (4) Healthcare professionals are aware of the key risk factors associated with post-stroke dementia. (5) Over half of all healthcare professionals thought stroke physicians should inform the patient about risk of dementia. Over 80% of respondents said they would inform the patient's GP. (6) The majority of healthcare professionals thought patients should be informed around 1-6 months after stroke, but it should be dependent on the individual patient. (7) If a prediction tool was available, healthcare professionals have a lower threshold of risk to inform the patient's GP compared to the patient.

### Existing research

Risk prediction models for dementia exist, but many have not been externally validated and the performance of these models varies depending on the cohort it is tested on.^22^ Prognostic models, specific to the stroke population are being developed, however, international guidelines on post-stroke cognitive impairment recognise that there are currently no tools suitable for use in clinical practice.^7^^,^^27^ Should a risk prediction tool be suitable for use in clinical practice, it is important that the ethics of discussing risk of dementia are carefully considered. A previous study explored the feasibility of using a prediction tool for dementia as part of follow-up care for people who have a stroke.^8^ Whilst patients and carers thought that knowing their risk of dementia may be helpful to prepare for the future and help them to feel supported, they had concerns about the impact it would have on their recovery. ^8^ There is clearly a balance between educating stroke patients about risk of dementia and the support that is available without causing them distress.

Cognitive problems following stroke are a key concern to stroke survivors.^3^ Stroke patients describe being fearful of receiving a dementia diagnosis.^15^ It is therefore important that this information is communicated in a supportive way by healthcare professionals who are knowledgeable about the care and support that is available. Our findings conform with the findings of previous research suggesting that healthcare professionals find it challenging to have a discussion about memory issues both at acute stroke and during follow-up.^17^

Several respondents reported they would discuss risk of dementia if the patient/carer asked. Nevertheless, if patients are not educated about the increased risk of dementia following a stroke, they are unlikely to ask about the support that is available and may not recognise the symptoms of dementia. Stroke survivors have reported challenges with accessing support for memory problems.^15^ Informing patients at the time of stroke about the support that is available, or who to contact, may help.

Healthcare professionals have highlighted gaps in care for people with post-stroke cognitive problems.^17^ Similarly, in our study, we found that there was considerable uncertainty around which healthcare professionals should inform patients about risk of dementia and when was the most appropriate time to discuss dementia risk. The majority of healthcare professionals thought patients should be informed about risk of dementia between 1-6 months following a stroke, with some suggesting that cognition should be monitored over time. However, many patients do not receive a follow-up appointment with the rehabilitation team, meaning patients would need to proactively contact their GP if they have concerns.

Whilst guidelines for post-stroke cognitive impairment exist, there is no standard care-pathway for patients at risk of post-stroke cognitive problems.^27^ Given the limited opportunity to discuss post-stroke cognitive problems at follow-up, this UK wide survey explored the feasibility of discussing risk of dementia at the time of a stroke, whilst the patient is already receiving medical help.

### Implications for clinical practice

Increasing healthcare professionals’ awareness of the risk factors for post-stroke dementia and providing them with training in how to have conversations about post-stroke cognition may encourage them to discuss cognitive impairment with patients when appropriate. The cost of dementia care in the UK is estimated to rise to £94.1 billion in 2040.^28^ Educating patients with acute stroke may help patients and relatives to look out for signs or symptoms of cognitive impairment, and to consider behavioural changes to modify risk factors. Identifying people who are at risk of post-stroke cognitive problems may provide an opportunity for rehabilitation specialists to screen for memory impairment as part of physical and occupational therapy after stroke. Having a care-pathway in place for people who have a stroke that provides specialised care for patients at risk of cognitive problems, at regular time points after their stroke, may help people with concerns around cognition to access support for memory problems and enable earlier detection of dementia.

### Implications for research

Our study found that healthcare professionals are aware of the risk factors for post-stroke dementia. Stroke patients, who present with key risk factors for post-stroke dementia, at the time of stroke, could be targeted to clinical trials that aim to identify treatments for dementia. Further studies co-designed with stroke survivors and their families, and the multidisciplinary team who care for them, could target whether and how to raise awareness of potential cognitive consequences of stroke.

### Strengths and limitations

A multidisciplinary team of healthcare professionals are involved in caring for patients with stroke. A strength of this survey is that it sought views from all healthcare professionals who care for patients with stroke throughout the UK. However, responses were not representative of all healthcare professionals who care for patients with stroke. We received a disproportionate number of responses from healthcare professionals working in Scotland, and few responses from certain allied health professionals such as Occupational Therapists and Physiotherapists.

The survey used self-reported measures, which may not reflect what happens in clinical practice. However, responses to this survey were anonymous which should have encouraged respondents to answer honestly. As we distributed the survey via social media, we were unable to calculate how many healthcare professionals received an invitation to the survey, nor could we formally verify the respondents are healthcare professionals. We also did not research the views of patients with stroke and carers.

## Conclusions and future research

There may be benefits and risks to informing patients with acute stroke about their future risk of dementia. We need to understand how to clearly and appropriately communicate this prognostic information to patients and explore how follow up care could be tailored for patients at high risk of post stroke dementia.

## Declaration of Competing Interest

None.

## Appendix Supplementary materials

Image, application 1

Image, application 2
