# Supplementary material for: Informing Patients with Acute Stroke About their Risk of Dementia: A Survey of UK Healthcare Professionals
Source: J Stroke Cerebrovasc Dis. 2022 Mar;31(3):None. doi: 10.1016/j.jstrokecerebrovasdis.2021.106279 (PMC8849345; doi:10.1016/j.jstrokecerebrovasdis.2021.106279)
Supplement: Supplementary file 1 [file mmc1.docx]

**Supplementary material**

**Informing patients with acute stroke about their risk of dementia: A survey of UK healthcare professionals**

**Appendix 1: Survey questions and response options**

|  | **Questions** | **Response options** |
| --- | --- | --- |
| **1.** | Which country do you currently work in? | Scotland, England, Northern Ireland, Wales |
| **2.** | What is your background? | Stroke physician, Stroke specialist nurse, Occupational therapist, Physiotherapist, Speech and language therapist, Clinical psychologist or clinical neuropsychologist, Neurologist, Geriatrician, General practitioner, Other (free text response) |
| **3.** | Where do you usually work with stroke patients? | Stroke unit, Acute assessment ward, Emergency Department, GP Practice, Other (free text response) |
| **4.** | How long have you been working with stroke patients? | Less than 1 year, 1-5 years, 6-10 years, 11-15 years, 16-20 years, 21+ years |
| **5.** | **Imagine that you see a patient who had a stroke 5 days ago. You are considering whether this patient is at risk of developing dementia within the next year. Which of these factors would make you concerned that this person has a high future risk of dementia? (Select all options that apply)** | |
|  | Demographic factors | Age 65 years or older, Age under 65 years, Male, Female, Low educational level, High educational level, Social deprivation, Ethnicity: black and minority ethnic groups, Ethnicity: white |
|  | Pre-stroke clinical factors | Had previous stroke, Had previous TIA, History of high blood pressure, History of diabetes, History of high cholesterol, Asthma, History of ischemic heart disease, Cancer, History of high alcohol intake, Current or previous smoker |
|  | Neuroimaging factors observed on an acute stroke CT scan | White matter hyperintensities, Silent brain infarct(s), Global atrophy, Large infarct(s), Presence of old infarct(s), Tumour |
|  | Factors relating to acute stroke | Ischemic stroke, Hemorrhagic stroke, Severity of stroke, Had delirium during the patient’s hospital stay |
|  | Other | None of the above mentioned factors, Unsure, Other (free text response) |
| **6.** | **Think about the patients with acute stroke that you have seen in the past year.**  **(Acute stroke period = within 2 weeks of stroke)** | |
| **a.** | How often did you discuss the risk of developing DEMENTIA with patients with acute stroke? | All of the time, Most of the time, Sometimes, Rarely, Never, I haven’t seen any patients within the acute stroke period |
| **b.** | How often did you discuss the risk of a CONTINUOUS DECLINE IN COGNITION with patients with acute stroke? | All of the time, Most of the time, Sometimes, Rarely, Never, I haven’t seen any patients within the acute stroke period |
| **c.** | How often did patients with acute stroke ask about the risk of developing DEMENTIA? | All of the time, Most of the time, Sometimes, Rarely, Never, I haven’t seen any patients within the acute stroke period |
| **d.** | How often did patients with acute stroke ask about the risk of a CONTINUOUS DECLINE IN COGNITION? | All of the time, Most of the time, Sometimes, Rarely, Never, I haven’t seen any patients within the acute stroke period |
| **e.** | How often did relatives/carers ask about the risk of the patient developing DEMENTIA? | All of the time, Most of the time, Sometimes, Rarely, Never, I haven’t seen any patients within the acute stroke period |
| **f.** | How often did relatives/carers ask about the risk of the patient having a CONTINUOUS DECLINE IN COGNITION? | All of the time, Most of the time, Sometimes, Rarely, Never, I haven’t seen any patients within the acute stroke period |
| **g.** | Please provide any additional comments regarding your answers to the above questions | Free text response |
| **7.** | **Please complete the following statement (select all options that apply)** | |
|  | I discuss the risk of post-stroke dementia: | With all patients with acute stroke, With patients with acute stroke that I think have a high risk of developing dementia, If the patient with acute stroke or their carer asks about post-stroke dementia, I have never discussed post-stroke dementia with a patient with acute stroke, Other (free text response) |
| **8.** | **How likely are you to discuss the risk of dementia with the following patients who had their stroke/TIA 5 days ago?** | |
| **a.** | A patient who had a TIA | Extremely likely, Likely, Neutral, Unlikely, Extremely unlikely |
| **b.** | A patient who had a mild stroke (NIHSS 1-4) | Extremely likely, Likely, Neutral, Unlikely, Extremely unlikely |
| **c.** | A patient who had a moderate stroke (NIHSS score 5-15) | Extremely likely, Likely, Neutral, Unlikely, Extremely unlikely |
| **d.** | A patient who had a moderate to severe stroke (NIHSS 16-20) | Extremely likely, Likely, Neutral, Unlikely, Extremely unlikely |
| **e.** | A patient who had a severe stroke (NIHSS 21-42) | Extremely likely, Likely, Neutral, Unlikely, Extremely unlikely |
| **9.** | **Imagine that you are able to identify which patients YOU think are at high risk of developing dementia within the next year. Please respond to the following statements regarding patients with acute stroke:** | |
| **a.** | I would discuss the risk of dementia with the patient with acute stroke | Strongly agree, Agree, Neither agree nor disagree, Disagree, Strongly disagree |
| **b.** | I would make the patient's GP aware that the patient is at high risk of post-stroke dementia | Strongly agree, Agree, Neither agree nor disagree, Disagree, Strongly disagree |
| **c.** | I would feel confident discussing the risk of post-stroke dementia with the patient | Strongly agree, Agree, Neither agree nor disagree, Disagree, Strongly disagree |
| **d.** | I think the patient would benefit from knowing they are at high risk of post-stroke dementia | Strongly agree, Agree, Neither agree nor disagree, Disagree, Strongly disagree |
| **e.** | I think the patient's family/carers would benefit from knowing the patient is at high risk of post-stroke dementia | Strongly agree, Agree, Neither agree nor disagree, Disagree, Strongly disagree |
| **f.** | Please provide any additional comments regarding your answers to the above questions | Free text response |
| **10.** | **Imagine that you are able to identify which patients YOU think are at high risk of developing dementia within the next year.** | |
|  | When would you choose to inform the stroke patient about their risk of post-stroke dementia? | Within 2 weeks of their stroke, 1 month after their stroke, 3 months after their stroke, 6 months after their stroke, I would not inform the patient, Other (free text response) |
| **11.** | **Think about your answer to the previous question.** | |
|  | At this point in time, who do you think should inform the patient about their risk of post-stroke dementia? | Stroke physician, Stroke specialist nurse, Occupational therapist, Physiotherapist, Speech and language therapist, Clinical psychologist or Clinical neuropsychologist, General Practitioner, No one should inform the patient, Other (free text response) |
| **12.** | **Imagine that you see a patient who has had a stroke 5 days ago. You input the patient's information into a highly accurate risk prediction tool and calculate their risk of developing post-stroke dementia within the next year.** | |
| **a.** | At which level of risk would you discuss post-stroke dementia with the patient? | 0-20% risk of post-stroke dementia, 21-40% risk of post-stroke dementia, 41-60% risk of post-stroke dementia, 61-80% risk of post-stroke dementia, 81-100% risk of post-stroke dementia |
| **b.** | At which level of risk would you make the patient's GP aware of the risk of post-stroke dementia | 0-20% risk of post-stroke dementia, 21-40% risk of post-stroke dementia, 41-60% risk of post-stroke dementia, 61-80% risk of post-stroke dementia, 81-100% risk of post-stroke dementia |
| **13.** | **Please provide any further comments you may have about discussing the risk of post-stroke dementia at the point of a stroke.** | |
|  | Free text response | |

| **Appendix 2: Imagine that you are able to identify which patients you think are at high risk of developing dementia within the next year. Please respond to the following statements regarding patients with acute stroke:** | | | |
| --- | --- | --- | --- |
| **Question** | **Strongly agree/**  **Agree**  **N (%)** | **Strongly disagree/**  **Disagree**  **N (%)** | **Neither agree nor disagree**  **N (%)** |
| I think the patient would benefit from knowing they are at high risk of post-stroke dementia | 34 (57) | 7 (12) | 19 (32) |
| I think the patient's family/carers would benefit from knowing the patient is at high risk of post-stroke dementia | 45 (75) | 3 (5) | 12 (20) |

| **Appendix 3: Please complete the following statement (select all options that apply).** | |
| --- | --- |
| **I discuss the risk of post-stroke dementia:** | **Agree**  **N (%)** |
| With all patients with acute stroke | 1 (2) |
| With patients with acute stroke that I think have a high risk of developing dementia | 7 (12) |
| If the patient with acute stroke or their carer asks about post-stroke dementia | 30 (50) |
| I have never discussed post-stroke dementia with a patient with acute stroke | 22 (37) |
| Other (free text response) | 2 (3) |

| **Appendix 4: Think about the patients with acute stroke that you have seen in the past year.** | | | | |
| --- | --- | --- | --- | --- |
| **Question** | **All/Most of the time**  **N (%)** | **Sometimes**  **N (%)** | **Rarely/Never**  **N (%)** | **I haven’t seen any patients within the acute stroke period**  **N (%)*** |
| How often did you discuss the risk of developing DEMENTIA with patients with acute stroke? | 2 (3) | 4 (7) | 47 (78) | 7 (12) |
| How often did you discuss the risk of a CONTINUOUS DECLINE IN COGNITION with patients with acute stroke? | 2 (3) | 14 (23) | 37 (62) | 7 (12) |
| How often did patients with acute stroke ask about the risk of developing DEMENTIA? | 2 (3) | 12 (20) | 40 (67) | 6 (10) |
| How often did patients with acute stroke ask about the risk of a CONTINUOUS DECLINE IN COGNITION? | 2 (3) | 10 (17) | 42 (70) | 6 (10) |
| How often did relatives/carers ask about the risk of the patient developing DEMENTIA? | 0 (0) | 23 (38) | 32 (53) | 5 (8) |
| How often did relatives/carers ask about the risk of the patient having a CONTINUOUS DECLINE IN COGNITION? | 2 (3) | 16 (27) | 37 (62) | 5 (8) |
| *The number of respondents that said they had not seen any patients within the acute stroke patients varied for each question | | | | |

| **Appendix 5: How likely are you to discuss the risk of dementia with the following patients who had their stroke/TIA 5 days ago?** | | | |
| --- | --- | --- | --- |
| **Question** | **Extremely likely/**  **Likely**  **N (%)** | **Extremely unlikely/**  **Unlikely**  **N (%)** | **Neutral**  **N (%)** |
| A patient who had a TIA | 2 (3) | 51 (85) | 7 (12) |
| A patient who had a mild stroke (NIHSS 1-4) | 4 (7) | 51 (85) | 5 (8) |
| A patient who had a moderate stroke (NIHSS score 5-15) | 7 (12) | 36 (60) | 17 (28) |
| A patient who had a moderate to severe stroke (NIHSS 16-20) | 17 (28) | 30 (50) | 13 (22) |
| A patient who had a severe stroke (NIHSS 21-42) | 16 (27) | 33 (55) | 11 (18) |

**Appendix 6: When would you choose to inform the stroke patient about their risk of post-stroke dementia?**


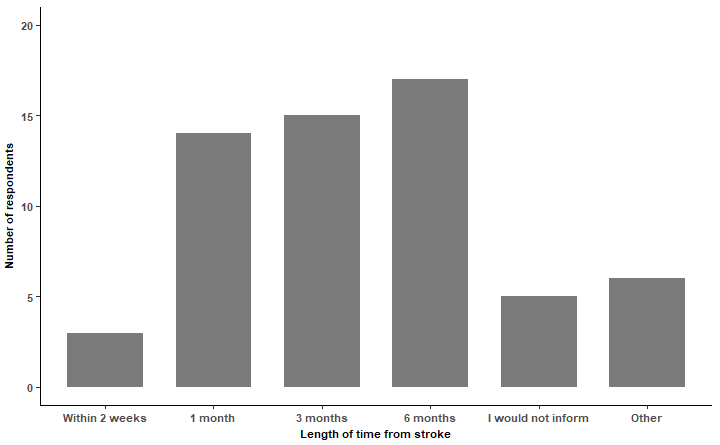


| **Appendix 7: Think about your answer to the previous question. At this point in time, who do you think should inform the patient about their risk of post-stroke dementia?** | |
| --- | --- |
| **Question** | **N (%)** |
| Stroke physician | 32 (53) |
| General Practitioner | 7 (12) |
| Stroke specialist nurse | 6 (10) |
| Clinical psychologist or clinical neuropsychologist | 3 (5) |
| Occupational therapist | 3 (5) |
| Physiotherapist | 0 (0) |
| Speech and language therapist | 0 (0) |
| No one should inform the patient | 0 (0) |
| Other (free text response) | 9 (15) |

| **Appendix 8: Imagine that you are able to identify which patients you think are at high risk of developing dementia within the next year. Please respond to the following statements regarding patients with acute stroke.** | | | |
| --- | --- | --- | --- |
| **Question** | **Strongly agree/**  **Agree**  **N (%)** | **Strongly disagree/**  **Disagree**  **N (%)** | **Neither agree nor disagree**  **N (%)** |
| I would discuss the risk of dementia with the patient with acute stroke | 29 (48) | 16 (27) | 15 (25) |
| I would make the patient’s GP aware that the patient is at high risk of post-stroke dementia | 40 (67) | 7 (12) | 13 (22) |
| I would feel confident discussing the risk of post-stroke dementia with the patient | 27 (45) | 15 (25) | 18 (30) |

| **Appendix 9: Free text responses - Focus of acute stroke discussions in current clinical practice** |
| --- |
| “During the acute stroke time it is not the best moment to discuss future possible dementia risk, when survival is the first concern.” |
| “Patient and family assumptions tend to be that person will get better after a stroke, rather than worse” |
| “I don't think the acute phase is necessarily the best setting. During this phase, the focus is on recovery and rehabilitation.” |
| “Often talk about physical disability and cognitive impact, processing, apathy etc.” |
| “Most questions and discussion about cognition was either regarding acute cognitive changes post-stroke, or pre-stroke cognitive decline which may or may not have been exacerbated by the stroke” |
| “People think that the cognitive effects : especially memory loss might be dementia and are relieved when the acute stroke effects are explained” |
| “Within the acute period (up to a few weeks post stroke) I tend to focus on acute care investigation and sec prevention.” |
| “May be too much information to process and accept in the very early days for some patients and families” |
| “I think the focus should remain on rehabilitation at this point.” |
| “need to focus on acceptance of Stroke, and adaptation and Rehab in first instance” |

| **Appendix 10: Free text responses - Factors that would influence whether healthcare professionals discuss dementia, at the time of stroke** |
| --- |
| “if patient is already showing signs of cognitive impairment or delirium” |
| “I would have these discussions if initiated by the patient or their caregivers” |
| “educating families about the impact of dementia post stroke is important” |
| “Conversation often pre-empted by the clinical course of the patient. If delirium at time of presentation or slow to progress or engage in rehab” |
| “If the patient already has cognitive decline and CT scan indicates possibility. Delirium as inpatient and part of general review” |
| “All depends on patient’s current cognition” |
| “I think if patients or families ask we should be honest” |
| “it may be better to stay positive regarding plasticity and recovery” |
| “If we had treatments that could prevent or delay post stroke cognitive decline” |
| “depends if you believe risk is modifiable, I think that lifestyle factors and cognitive interventions help (but appreciate there is no great evidence)“ |
| “unless asked” |
| “I am not saying that education regarding dementia following stroke should not be provided” |
| “unless there is already a clear pattern of decline / existing diagnosis, or someone already has a presentation which is more typical in dementia than stroke” |
| “Not much point discussing risk without some form of positive action to accompany it” |
| “if by giving the information, there is a change in lifestyle factors, that HAS to translate to a reduction in incidence otherwise are we not just giving people a spectre to live under for longer? I’m not sure the science IS necessarily there in long terms studies that changing risk factors reduces the incidence of dementia” |
| “how this will aid the patient” |

| **Appendix 11: Free text responses - Patient/carer related factors** |
| --- |
| “I suspect most people living with dementia and their caregivers would struggle to separate a decline in thinking skills / dementia. There is so much information sharing in the acute period that adding discussions about dementia into the mix maybe too much for most people” |
| “Even then may not be the right moment for anyone with low or vulnerable mood.“ |
| “This is of course patient dependent” |
| “May be too much information to process and accept in the very early days for some patients and families” |
| “I feel this is very much patient specific and their readiness to know the information. Some people may already have high levels of anxiety and the additional information of potentially developing dementia would add further distress at an already very distressing point in their life. “ |
| “The issue is the diagnosis of dementia would be difficult to accept especially with relatives.“ |
| “look at each person as an individual, it’s the only way, some may not wish to know what’s ahead “ |
| “it may be very difficult for patients and their relatives to take on board and retain information about vascular dementia“ |
| “I am also concerned about the psychological effects of discussing theoretical future risk in the context of a life changing acute stroke event “ |
| “Routine post stroke follow-up advice. However the risks of being pessimistic is that a patient will be depressed.“ |
| “Otherwise it’s just more bad news on top of an already distressing situation.” |
| “I work with patients who have communication problems after stroke, for many of these it would be challenging to discuss risk factors due to communication problems and may make them more worried at a time when they are already dealing with the impact of a stroke” |

| **Appendix 12: Free text responses – Post-stroke care-pathway** |
| --- |
| “There is so much information sharing in the acute period that adding discussions about dementia into the mix maybe too much for most people - I would have these discussions if initiated by the patient or their caregivers but otherwise would wait till a later follow-up “ |
| “immediately after stroke lots of my patients have cog issues that get better over the weeks. I tend to defer any discussion about cog prognosis till I see them back in the Outpatient Department “ |
| “I think this information is perhaps better delivered sometime following the acute stroke phase” |
| “conversation often pre-empted by the clinical course of the patient. If delirium at time of presentation or slow to progress or engage in rehab.” |
| “I find these questions and conversations arise at follow up. Within the acute period (up to a few weeks post stroke) I tend to focus on acute care investigation and sec prevention. Patients get a lot of info at this stage - this is a complex conversation re cognition best done when the acute period has settled” |
| “I tend to discuss this with patients when referred by the six month follow up service if they are reporting deteriorating cognition and radiologist reports suggest severe small vessel disease. Here, I tend to do a TOPF/WMS-IV assessment and depending upon this will refer to old age psychiatry for further assessment.” |
| “Once they are home and starting to rebuild their life and we are discharging them from therapy services we should give them ongoing advice to reduce risks. I.e. keeping active body and mind, healthy lifestyle, ?? medications.” |
| “Not fair to add workload to GPs - if the stroke team raise the issue, the stroke team need to deal with it“ |
| “I don't think this should be discussed at the acute stage but perhaps at follow up clinic.” |
| “If the patient is a stroke survivor, then during follow-up year it becomes relevant.” |
| “That discussion may be more beneficial / more likely to be taken in, in the less acute stage.” |
| “I don't think the acute phase (2 weeks following) is the correct timing. I think the focus should remain on rehabilitation at this point. I am not saying that education regarding dementia following stroke should not be provided” |
| “I feel that doctors are better placed to talk about risk of future dementia than me (an SLT). I often don't even see people's brain scans.” |
| “I would discuss this not at the time of the acute stroke however, but at OP follow up post discharge (or during inpatient stay for rehab).” |
| “I do not think this is the best time to focus on a ‘risk’ based conversation- best on follow up” |
| “Acute stroke is not the time for this discussion. Many cognitive impairments post stroke improve. Potential decline risks need discussing at 6 month follow up point, after assessing cognition directly” |
| “6 week medical follow up whoever is holding clinic but likely stroke physician or senior stroke nurse” |
| “i think ideally a coordinated team approach to agree conversation and back up support as required” |
| “Whoever is experienced and knowledgeable enough to know when and how is the right way to do it.“ |
| “I think that a stroke physician or stroke specialist nurse may be well placed to advise on risk as they may see a patient on a more routine basis. I think that a clinical neuropsychologist would be well placed to assess patient's cognition and adaptive functioning at six months following their stroke to inform diagnosis, as necessary.” |
| “would a multidisciplinary approach be best, with medical and AHP input” |
| “depends on who feels comfortable to initiate the discussion and who the relationship the patient has with the clinician” |
| “whoever has ongoing involvement with their care” |
| “A dementia specialist, someone who has worked in memory clinics for 15 years.” |
| “I think this could be anyone from the stroke team that is following up the patient so long as they have the knowledge base to discuss this fully and its implications - most likely stroke physician, CNS, Specialist therapist with training in this area” |
| “This is not really something that has occurred to me before, although it may be something that I incorporate into my practice in the future, to aid anticipatory care planning.” |
| “I feel that too often patients are cognitively tested within days of having had a stroke and told that they have vascular dementia with no giving the patient time to improve which they often have done by the time they reach the memory clinic.” |
| “we are screening for dementia/delirium in most unscheduled older adult admissions - should do the same in stroke” |
| “Routine post stroke follow-up advice.” |
| “If the risk could be predicted then I think it is important to discuss with all patients and inform GP of these discussions to aid advanced planning” |
| “I think the GP should already be informed of the risks of dementia following stroke. There should perhaps be a pathway following stroke which involves assessment in a memory clinic following stroke a certain points in time (e.g. 6 months, 12 months and 18 months), to measure whether deterioration in cognition is occurring” |
| “I feel it could be a psychologist or specialist nurse as well.” |
| “I think these are hugely complex conversations - almost analogous to genetic counselling - they require time and thought not just statistics of risk” |
| “Too early, need to focus on acceptance of Stroke, and adaptation and Rehab in first instance. This belongs much further down the care pathway” |

| **Appendix 13: Free text responses - Using a risk prediction tool for post-stroke dementia** |
| --- |
| “I think we probably have enough science to be putting a relative risk / odds ratio typed algorithmic thing together? BUT what about lead-time bias if we can’t change the outcome?” |
| “Prediction tools for vascular dementia after stroke would be very useful. I think that in the acute phase (first two weeks) it may be very difficult for patients and their relatives to take on board and retain information about vascular dementia.” |
| “I am concerned about using prediction in the acute setting to potentially deny access to treatments if the patient is deemed 'poor prognosis' I am also concerned about the psychological effects of discussing theoretical future risk in the context of a life changing acute stroke event” |
| “Having access to tools to estimate rates of post stroke dementia would be extremely helpful in clinical practice” |
| “ ’Risk means risk over a certain time period. If the risk is 20% over the lifetime then that might be population norm. I would rather represent the information as an odds ratio relative to age-matched controls.” |
| “If the risk could be predicted then I think it is important to discuss with all patients and inform GP of these discussions to aid advanced planning” |
| “I don't like the concept of screening for a score if there is no specific intervention. Many scores have higher sensitivities and it is useful to know but this needs to be influenced as to how this will aid the patient. Will be guided by social structures and support mechanisms. would influence discharge plans and advance planning” |
| “I think these are hugely complex conversations - almost analogous to genetic counselling - they require time and thought not just statistics of risk” |
